# Should Adults Be Re Vaccinated?

**Published:** 1893-04-22

**Authors:** 


### The Hospital

An Institutional Journal of

Science, Medicine, Nursing, and Philanthropy.

*For Hospitals, Asylums, Infirmaries, Dispensaries, Medical Practitioners, Students, Nurses, and the Charitable Public.*

Vol. XIV.—No. 343.] April 22, 1893.

### Should Adults be Re-vaccinated?

Regular readers of The Hospital know that it has been our constant aim to free ourselves from prejudice on the subject of vaccination. The question of the re-vaccination of adults is now reopened for two reasons; first, because, according to Sir Edwin Galsworthy, we are threatened with a recrudescence of small-pox; and, secondly, because persistent attempts have been made during the past decade to bring this method of prophylaxis into general discredit. In what we have now to say there will be no attempt at special pleading. In our judgment the medical profession of the present day is no more bound to Jenner and Jennerism than modern China is bound to Confucius and Confucianism. If vaccination be worthless, let it be discredited; if it be a genuine prophylactic against small-pox, let its use be extended and established wherever small-pox may prevail.

Since bacteriology became a subject of universal stu y new weapons have been imported into the vaccination controversy. It is argued, for example, that the microbe of vaccine lymph is not identical with the microbe of the small-pox pustule; and, therefore, it is affirmed, "the one cannot act as a prophylactic against the other." Why not? The honey bee is not identical with humble bee; yet they are both bees; they both produce "comb"; moreover, they can both sting. Similarly the microbe of vaccine lymph, though not identical with the microbe of the small-pox pustule, is a microbe, and it is manifest that it belongs to a nearly-allied family, because it produces effects almost identical with those of the small-pox microbe when employed in similar quantities and circumstances. The two microbes both act as local irritants; they both set up general fever; they both produce "pocks" which are exceedingly similar, and run a very similar course. In a word, a man is a mason because he can build ho ses. He may be a bricklayer, but he is still a mason; he may speak the Belgian language instead of the English; but he is still a mason. So also, when we find two microbes producing such similar results as are produced by the vaccine microbe and the microbe of small-pox, we are entitled, we repeat, to conclude that they both belong to the same family, and that there is no more reason why one should not act as a prophylactic against the other than there is why the Belgian mason should not build up again a stone wall which the English mason has just thrown down.

But, says the objector, Jenner himself did not understand his own business of vaccination; he could not, for instance, make up his mind whether vaccine lymph had its origin in cowpox or in horse grease. That may be so. But we submit that whether Jenner himself knew little or much about vaccination is not to the point. The real question is, What does the medical profession of the present day know about it? Jenner was a pioneer and a discoverer. Many a Board School boy of this half of the century knows ten times more about the geography of Central Africa than Livingstone did, who was its first English explorer. But does that prove that Livingstone was a charlatan, or that Central Africa has no objective existence?

We do not, then, go back upon Jenner. Our trust is in modern investigation, modern experience, and modern straightforwardness. Those who are familiar with the younger members of the medical profession are well aware that there is no name and no reputation which is too sacred for investigation or too great for doubt. What are the kinds of facts which modern medical men have found when they have carried their torch of scientific inquiry into the secret places of the history of vaccination? They have found one grand series of negative facts which is patent to all the world. "Pockmarked" men and women, who were formerly as plentiful as blackberries, are now conspicuous by their absence. They are as rare as snow in summer or as sober minded anti-vaccinators.

But positive facts, statistics, constitute the real stronghold of the modern vaccinator. Now the parrot-minded person has somewhere heard it said that "statistics will prove anything"; and, parrot-like, his morning greeting is, "Statistics will prove anything"; and his evening adieu, "Statistics will prove anything." If this kind of trifling pleases him, so be it. But practical persons seek after practical things; and to practical persons there is no single argument so convincing as statistics, providing that they cover the ground adequately, and are accumulated by competent and disinterested persons. The questions we have to ask, then, are two: Do the statistics of vaccination cover sufficient ground? And have they been accumulated by competent persons? To these it is answered that the statistics cover nearly a century of time, and the whole of the civilised world. With regard to the competency of those who have accumulated them, there has never been but one opinion among persons of candour and common sense. Take, for example, the accumulators of vaccination statistics during the last quarter of a century. Who are they? Who have they been? The Registrar General for Great Britain, and similar state officials in other countries; the chief medical officials of the Local Government department; the medical superintendents of small-pox hospitals, and other medical and non-medical persons whose circumstances have given them special opportunities. Now nobody can deny that these persons are at least competent. Are they also disinterested? The answer is that they are bound to be disinterested, because there is every reason in the world why they should be disinterested, and not one single reason why they should not. If it be said that "as medical men they are bound up with Jenner and his fame"; the answer is a question: Why are they bound up with Jenner and his fame? What does the modern superintendent of a small-pox hospital care for Jenner and his fame? Why should he care for it? Above all, why should the Registrar General for Britain concern himself with Jenner's fame? Still less those heads of State departments who hold corresponding positions in other countries? The writer is a medical man, but he cares no more for Jenner's fame than he does for the fate of last year's swallows; and he is sure that in this the vast majority, at any rate of his younger medical brethren, are in the fullest and completest agreement with him.

To come, then, to the question at the head of this article, Should adults be re-vaccinated? The answer is emphatically, they should! The reasons can be put into a sentence or two. Vaccination protects children, but the protection gradually wears out. Before vaccination the chief mortality from small-pox was amongst children: since the compulsory enforcement of vaccination the mortality amongst well-vaccinated children has fallen by 80 per cent., whilst the chief mortality is now amongst grown-up people. Of grown-up people, soldiers and sailors in most civilised countries are compulsorily re-vaccinated; and amongst them the mortality from small-pox approximates to that amongst vaccinated children. On the other hand, after the tenth year of life protection begins decidedly to wear out; so that in the period between 1872-80 the rate of mortality from smallpox rose 59 per cent. between the ages of 15 and 25, 114 per cent. between the ages of 25 and 45, and 164 per cent. from 45 upwards. These facts speak for themselves. So far as present knowledge and experience show, vaccination has proved itself one of the greatest prophylactics the world has ever seen. We must live by what we know, not by what we hope to know. That is the verdict of universal science and sense.

